# Molecular characterization of β-lactamase genes in clinical isolates of carbapenem-resistant *Acinetobacter baumannii*

**DOI:** 10.1186/s12941-017-0248-3

**Published:** 2017-11-16

**Authors:** Kevin M. Raible, Bhaswati Sen, Nancy Law, Tiffany E. Bias, Christopher L. Emery, Garth D. Ehrlich, Suresh G. Joshi

**Affiliations:** 1 Center for Surgical Infections & Biofilms, Institute of Molecular Medicine and Infectious diseases, Drexel University College of Medicine, 245 N. 15th Street, Philadelphia, PA 19102 USA; 2 Center for Genomic Sciences, Institute of Molecular Medicine and Infectious diseases, Drexel University College of Medicine, 245 N. 15th Street, Philadelphia, PA 19102 USA; 3 Center for Advanced Microbial Processing, Institute of Molecular Medicine and Infectious Diseases, Drexel University College of Medicine, 245 N. 15th Street, Philadelphia, PA 19102 USA; 4 Department of Microbiology and Immunology, Drexel University College of Medicine, 245 N. 15th Street, Philadelphia, PA 19102 USA; 5 Department of Otolaryngology-Head and Neck Surgery, Drexel University College of Medicine, 245 N. 15th Street, Philadelphia, PA 19102 USA; 6 Present Address: Department of Pathology and Lab Medicine, Indiana University School of Medicine, Indianapolis, IN USA

**Keywords:** *Acinetobacter baumannii*, β-lactamase, Biofilm, Carbapenem resistance, Colonization, CRAB, Integrase, Integron cassette, Multiple drug resistance, Multilocus sequence typing, MLST, OXA-51, OXA-23, OXA-40, Plasmid

## Abstract

**Background:**

*Acinetobacter baumannii* is a nosocomial pathogen which is establishing as a major cause of morbidity and mortality within the healthcare community. The success of this pathogen is largely due to its ability to rapidly gain resistance to antimicrobial therapies and its capability to persist in an abiotic environment through the production of a biofilm. Our tertiary-care hospital has showed high incidence of carbapenem-resistant* Acinetobacter baumannii* (CRAB) isolates.

**Methods:**

In this study we explore both genotypic and phenotypic properties of 26 CRAB isolates: 16 isolates were collected from January 2010 to March 2011, and 10 were collected between February and May 2015.

**Results:**

We determined that all 26 CRAB isolates possessed multiple β-lactamase genes, including genes from Groups A, C, and D. Specifically, 42% of the isolates possesses the potentially plasmid-borne genes of OXA-23-like or OXA-40-like β-lactamase. The presence of mobile gene element integron cassettes and/or integrases in 88% of the isolates suggests a possible mechanism of dissemination of antibiotic resistance genes. Additionally, the location of insertion sequence (IS) IS*Aba*1 in promotor region of of the OXA-51-like, ADC-7, and ampC genes was confirmed. Multilocus sequence typing (MLST) demonstrated that all 26 CRAB isolates were either sequence type (ST)-229 or ST-2. Interestingly, ST-2 went from being the minority CRAB strain in the 2010–2011 isolates to the predominant strain in the 2015 isolates (from 32 to 90%). We show that the ST-2 strains have an enhanced ability to produce biofilms in comparison to the ST-229 strains, and this fact has potentially led to more successful colonization of the clinical environment over time.

**Conclusions:**

This study provides a longitudinal genetic and phenotypic survey of two CRAB sequence types, and suggests how their differing phenotypes may interact with the selective pressures of a hospital setting effecting strain dominance over a 5-year period.

**Electronic supplementary material:**

The online version of this article (10.1186/s12941-017-0248-3) contains supplementary material, which is available to authorized users.

## Background

*Acinetobacter baumannii* is an opportunistic pathogen that causes a variety of nosocomial infections resulting in considerable morbidity and mortality, and presents a particular threat to intensive care unit (ICU) patients [1–4]. The number of *A. baumannii* infections has been increasing globally over the past three decades; particularly with regard to infections involving multi-drug resistant (MDR) isolates. The rise of MDR bacterial strains is a major cause for concern among healthcare professionals and is prompting changes in both antibiotic regiments and hospital disinfection techniques [5–10]. Genomic studies exploring outbreaks of MDR *A. baumannii* from geographic regions distant from one another shows a high degree of similarity among strains, indicating that most outbreaks may be due to a limited number of clonal lines [11].

The ability of *A. baumannii* to rapidly gain resistance to antimicrobial compounds is an important population-level virulence factor [12] for the successful dissemination of the organism within and among health-care facilities [13, 14]. Most strains of *A. baumannii* are innately resistant to several classes of antibiotics such as first- and second-generation cephalosporins, chloramphenicol, and aminopenicillins; in addition, the ability to both accept exogenous genetic material and overexpress endogenous resistance genes has quickly resulted in the appearance of the MDR phenotype within multiple clonal lineages [6, 15]. Historically the drug of choice to treat severe *A. baumannii* infections has been carbapenems, the clinical use of both meropenem and imipenem increased dramatically in the last 15 years to treat newly emerging MDR strains [1]. Likely as a result of this selective pressure carbapenem-resistant *Acinetobacter baumannii* (CRAB) strains are being reported with greater frequency.

In addition to antibiotic resistance, the ability to adhere and produce biofilm on both biotic and abiotic surfaces has been shown to be a virulence factor in many clinical isolates of *A. baumannii* and other bacterial pathogens [16–19]. The ability of clinical isolates to adhere to lung epithelial cells which is a critical step in the establishment of lung infections, and have also been documented [20–22]. The organism survives even on abiotic surfaces for months, and numerous retrospective epidemiological studies have shown its ability to colonize medical equipment, furniture, and healthcare personnel; this phenomenon provides the organism the capability of causing outbreaks both within and among medical institutions [23–28].

In the present study, we characterize the carbapenem resistance genotypic profile, as well as the colonization-associated phenotypes of 26 CRAB isolates, collected between 2010 and 2015, from a tertiary care hospital in Philadelphia, Pennsylvania. By analyzing these data, we aimed to determine if there was a temporal trend in resistance or colonization among these clinical isolates.

## Methods

### Bacterial isolates and reference strains

This study included 26 clinical isolates of carbapenem-resistant [minimum inhibitory concentration of meropenem (MIC) ≥ 8 mg/L] isolated from Hahnemann Hospital of Drexel University College of Medicine, Philadelphia, PA. Sixteen of the isolates were collected between 2010 and 2011 and were previously partially described by Sen and Joshi [29], (old isolates) and 10 isolates were collected in 2015 (New Isolates) which have not been described to date. The isolates were randomly selected, based on their MIC to meropenem. *A. baumannii* isolates were identified using the Vitek2 system (BioMerieux Vitek Systems Inc., USA), and confirmed by a molecular technique of OXA-51 PCR. Clinical Laboratory Standards Institute (CLSI)—recommended quality control (QC) strains were used as reference strains when necessary. QC strains included: *A. baumannii* ATCC 19606, *A. baumannii* ATCC 17978; *Escherichia coli* ATCC-BAA-2452, NCTC 13476; *Klebsiella pneumoniae* ATCC-BAA-2146, *K. pneumoniae* ATCC-BAA-1705, *K. pneumoniae* NCTC13440, and *Enterobacter cloacae* NCTC 13464. Additional file 1: Table S1 shows the QC strains and the reference gene for which they were used. The study was approved by the Institutional Review Board (IRB) of Drexel University, and a waiver granted.

### Determination of minimum inhibitory concentration (MIC)

Carbapenem MIC results were obtained for each CRAB isolate using the VITEK 2 system and the results were confirmed by broth microdilution. All clinical breakpoints recommendations and broth microdilution protocols were followed as per Clinical Laboratory Standards Institute (CLSI) guidelines [30]. The antimicrobial agents tested were ampicillin/sulbactam, imipenem, meropenem, amikacin, levofloxacin, gentamicin, cefepime, tobramycin, trimethoprim/sulfamethoxazole and piperacillin. In routine, our hospital laboratory does not include and report polymyxin and tigecycline for this organism, and hence these agents were not included to study panel.

### Polymerase chain reaction (PCR) Amplification and DNA Sequencing

Using DNeasy Blood and Tissue kit (Qiagen, Valencia, CA), genomic DNA was isolated from bacterial cells. All PCR primers targeting resistance genes and mobile elements used in this study are listed in Additional file 2: Table S2. PCR was performed using Thermo Scientific Dream Taq Green PCR Master Mix (Thermo-Fisher Scientific, Inc, Waltham, MA) using conditions specified by the reference sources. The isolates were tested for each target gene two–four times. Negative controls were included with every PCR setup to monitor for carryover [31] and rule out nonspecific amplification. Appropriate positive control strains were used to confirm the validity of each PCR primer pair (Additional file 1: Table S1). Wherever an appropriate QC strain for a particular gene was unavailable the amplicon present with the approximate size of the predicted product was purified using described method and sequences analyzed, and gene identifications were made using NCBI nucleotide *BLA*ST tool (https://blast.ncbi.nlm.nih.gov/Blast/). The presence of IS elements (IS*Aba1*) upstream of target genes following the method described by Sen and Joshi [29].

### Meropenem-EDTA double disk synergy test (DDS)

DDS was performed as described previously by Yong et al. using 0.5 McFarland standards, and disks containing Meropenem (10 µg) alone; Meropenem (10 µg) plus 10 µL of 0.5 M EDTA; and 0.5 M EDTA alone. Appropriate control strains were included and synergistic results were interpreted as described [29, 32].

### Multi-locus sequence typing (MLST)

MLST assignments were determined using the scheme provided by Pasteur Institute MLST and Whole Genome MLST Database (https://www.pasteur.fr/mlst). MLST target genes, PCR primer sequences, and annealing temperatures were all adopted from the database and optimized. The resulting PCR amplicons were run on a 1% agarose gel to determine the amplicon size and quality, and the PCR product was purified using GeneJet PCR purification kit (ThermoFisher), and sent to GeneWiz, Inc. (GeneWiz) for Sanger sequencing. The allelic identification of the sequences and strain determination was performed using the Pasteur MLST database (http://www.pubmlst.org/abaumannii/).

### Biofilm formation assay

Biofilm production was determined using 96-well polystyrene plates as described by O’Toole et al., with minor modifications [33]. Briefly, overnight cultures were grown in LB broth at 37 °C in an aerobic incubator. The following day, 200 µL LB broth diluted cultures (1:100) were added to multiple wells and the plates incubated at 37 °C in a static aerobic incubator for either 24 or 48 h. At the appropriate time the optical density was read at 595 nm, then each well received 30 µL of 0.1% Crystal Violet solution to incubate for 1 h at room temperature. After incubation, the plates were washed with tap water three times and allowed to dry at room temperature for 1 h. Then 250 µL of ethanol:acetone (4:1) solution was added to each well to solubilize the remaining stain. The optical density was then read at 570 nm, and the biofilm production was quantified by taking the OD_570_:OD_590_ ratio. The ratio was corrected with negative control (LB alone), and biofilm forming capability of the isolates considered if the OD_570_:OD_590_ ratio was a positive value. Experiments were run in two separate assays with a total of sixteen replicates each.

## Results

### Characterization of isolates

A total of 26 CRAB isolates were analyzed in this study, all collected from Hahnemann Hospital. Sixteen isolates were collected during 2010–2011 (old isolates), and the additional 10 isolates were collected in 2015 (new isolates). The relevant descriptive features are summarized in Table 1. The average age of all the patients from which the isolates were collected was 50.1 (± 17.7) years, with a range of 19–84 years. Of the 26 total patients 15 (57%) were male. A total of 14 (54%) patients were admitted in the ICU during the time of collection, and the other 12 (46%) patients were being treated for various conditions in non-ICU in-patient rooms in the hospital. A total of 12 (43%) patients’ infections were considered nosocomial in origin, in which the first positive *A. baumannii* culture was reported ≥ 48 h post-admission [34].

**Table 1:** Demographics of carbapenem-resistant *A. baumannii* positive patients

Features	2010–2011 isolates	2015 isolates
Number of isolates	16	10
Average age of patient (yr)	47.8 ± 19.1	55.6 ± 15.1
Male (%)	62	50
ICU patients (%)	37	50
Nosocomial infections (%)	37	60

### Antibiotic susceptibility

The findings of the antibiotic susceptibility testing for all isolates are showed in Additional file 3: Table S3. The antibiogram patterns of all 26 CRAB isolates by isolate groups (2010–2011 versus 2015), clinical source, and MLST sequence types are presented in Table 2. All 26 (100%) of the CRAB isolates met the clinical definition of the MDR *A. baumannii* phenotype, defined as being non-susceptible to at least 3 classes of antibiotics [34]. All isolates (100%) were resistant or intermediately resistant to carbapenems (meropenem and imipenem), as well as Levofloxacin (fluoroquinolone). More than 80% of the isolates were resistant or intermediately resistant to aminoglycosides, cephalosporins, extended-spectrum penicillins, and folate inhibitors.

**Table 2:** Antibiogram showing percent resistant isolates of different isolate groups (old and new, clinical source, and clonal lineage) against tested antimicrobial agents

	Ampicillin/sulbact (%)	Imipinem (%)	Meropenem (%)	Amikacin (%)	Levofloxacin (%)	Gentamicin (%)	Cefepime (%)	Tobramicin (%)	Trimeth-Sulfametho (%)	Piperacilin (%)
Old (n = 16)	68	100	100	50	100	93	93	88	93	93
New (n = 10)	40	100	100	100	100	60	100	10	90	100
Urine (n = 3)	66	100	100	33	100	100	100	100	100	100
Sp (n = 10)	90	100	100	70	100	100	100	60	90	100
Blood (n = 3)	66	100	100	66	100	100	100	33	100	100
Tx/Wound (n = 10)	40	100	100	80	100	50	90	40	90	90
#229 (n = 12)	75	100	100	50	100	92	100	83	83	92
#2 (n = 14)	57	100	100	85	100	71	92	28	100	100

The antimicrobial agents included were ampicillin/sulbactam, imipenem, meropenem, amikacin, levofloxacin, gentamicin, cefepime, tobramycin, trimethoprim/sulfamethoxazole and piperacillin

### Multi-locus sequence typing (MLST)

MLST was performed for all 26 CRAB isolates as described in Methods. A total of 12 (46%) isolates were determined to be ST-229 and 14 (54%) were determined to be ST-2. In the 2010–2011 isolate group, 11 (68%) of the 16 isolates were determined to be members of ST-229, while the remaining 5 (32%) isolates were identified as ST-2. In the 2015 isolate group, 9 (90%) of the isolates were identified as strain 2, and only 1 (10%) were determined to be ST 229 (Additional file 4: Figure S1). The isolates and their MLST sequence types are summarized in Table 3.

**Table 3:** PCR results for 26 CRAB isolates

Isolate	Source	MIC	MLST Type	TEM	SHV	PER	KPC	VEB	CTX	MBL	EDTA DDS	ADC-7	ISaba1 + ADC-7	ampC	ISaba1 + ampC	OXA-23	OXA-24	OXA-40	OXA-51	ISaba1 + OXA-51	OXA-58	ICA1	int123
22	Sp	16	229	+	+	–	–	–	–	–	–	–	–	+	+	–	–	–	+	+	–	+	+
25	Sp	16	229	+	+	–	–	–	–	–	–	+	+	+	+	–	–	–	+	+	–	+	+
28	T	8	2	+	–	–	–	–	–	–	–	+	+	+	+	–	–	–	+	+	–	–	+
32	U	32	229	+	+	–	–	–	–	–	–	+	+	+	+	–	–	–	+	+	–	+	+
39	Sp	16	229	+	+	–	–	–	–	–	–	+	+	+	+	–	–	–	+	+	–	+	+
42	T	16	229	+	+	–	–	–	–	–	–	+	+	+	+	–	–	–	+	+	–	+	+
51	T	16	229	+	+	–	–	–	–	–	–	+	+	+	+	–	–	–	+	+	–	+	+
52	U	16	229	+	+	–	–	–	–	–	–	+	+	+	–	–	–	–	+	+	–	+	+
56	U	32	229	+	+	–	–	–	–	–	–	+	+	+	+	–	–	–	+	+	–	+	+
57	U	8	2	+	–	–	–	–	–	–	–	–	–	+	+	–	–	–	+	+	–	–	+
58	T	> 256	2	+	–	–	–	–	–	–	–	+	+	+	–	–	–	+	+	–	–	–	–
61	U	16	229	+	+	–	–	–	–	–	–	–	–	+	+	–	–	–	+	+	–	+	+
62	Sp	16	229	+	+	–	–	–	–	–	–	–	–	+	+	–	–	–	+	+	–	+	+
64	T	32	2	+	–	–	–	–	–	–	–	+	+	+	+	+	–	–	+	+	–	–	–
65	B	16	2	+	+	–	–	–	–	–	–	–	–	+	+	–	–	–	+	+	–	+	+
68	Sp	8	229	+	–	–	–	–	–	–	–	+	+	+	+	–	–	–	+	+	–	–	+
220	T	32	2	+	–	–	–	–	–	–	–	–	–	+	+	+	–	–	+	+	–	–	+
222	B	64	2	+	–	–	–	–	–	–	–	+	+	+	+	+	–	–	+	+	–	–	+
223	Sp	32	2	+	–	–	–	–	–	–	–	+	+	+	+	+	–	–	+	+	–	+	+
224	T	64	229	+	+	–	–	–	–	–	–	+	+	+	+	–	–	–	+	+	–	+	+
225	T	32	2	+	–	–	–	–	–	–	–	+	+	+	+	+	–	–	+	+	–	–	+
226	T	32	2	+	–	–	–	–	–	–	–	+	+	+	+	+	–	–	+	+	–	+	+
229	Sp	32	2	+	–	–	–	–	–	–	–	+	+	+	+	+	–	–	+	+	–	+	+
231	Sp	64	2	+	–	–	–	–	–	–	–	+	+	+	+	+	–	–	+	+	–	–	+
232	T	> 256	2	+	–	–	–	–	–	–	–	+	+	+	+	–	–	+	+	+	–	–	+
235	T	32	2	+	–	–	–	–	–	–	–	+	+	+	+	+	–	–	+	+	–	–	+

Primer sets targeting β-lactamase genes (displayed by Ambler class), insertion sequence (IS*Aba*1), and mobile gene elements. Source (*Sp* sputum, *T* tissue/wound, *U* urine, *B* blood); *MIC* meropenem (≤ 2 μg/mL = Sensitive, ≥ 8 μg/mL = Resistant); KPC (KPC subtypes 1, 2, 5, 10); CTX (CTX-M subtypes 1, 2 8, 9, 25); *MBL* metallo-β-lactamase (DIM, GIM, IMP, NDM, SIM, SPM, VIM); EDTA *DDS* double disk synergy assay, *ICA1* class 1 integron cassette array, *int*123, conserved region of integrases 1, 2, and 3. All PCR reactions run in duplicate with proper control strains

### Extended-spectrum β-lactamase (ESBL) genotypic analysis

**Fig. 1:**
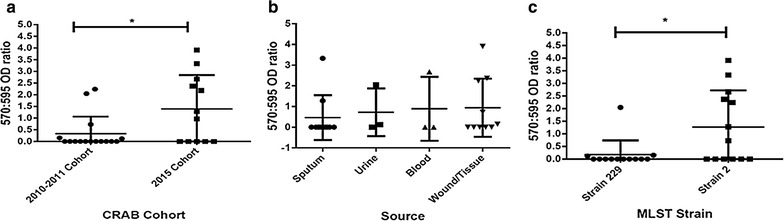
Biofilm production of CRAB isolates at 24 h (48 h data not shown) in 96-well polystyrene plate using Crystal Violet staining method. **a** Biofilm production with isolates grouped as old and new isolates (*p = 0.03). **b** Isolates grouped by clinical source exhibited non-significant value, may be due to lack of clonal heterogeneity. **c** CRAB isolates showing biofilm production, grouped by MLST sequence type/strain type (*p = 0.04)

Table 3 summarizes all the ESBL genes explored in this study. All CRAB isolates, and *A. baumannii* controls carried the Group D oxacillinase *bla*_OXA-51-like_ gene (Additional file 4: Figure S1), while no isolates contained the *bla*_OXA-24_ or *bla*_OXA-58-like_ gene. Two isolates, one from the 2010–2011 isolate group and one from the 2015 isolate group, possessed the *bla*_OXA-40-like_ gene (Fig. 1). Both *bla*_OXA-40-like_ positive isolates had the same MLST sequence type with a similar genotypic profile. Similarly, 9 (34%) isolates had *bla*_OXA-23-like_ genes (one from the 2010–2011 isolates and eight from the 2015 isolates) had the similar genotypic profiles. All of the CRAB isolates contained the Group A β-lactamase gene *bla*_TEM_, and 11 (42%) of the isolates had *bla*_SHV_. The *bla*_SHV_ positive isolates were all ST-229, suggesting a similar clonal linage. All of the isolates were negative for other Group A β-lactamase genes including *bla*_PER_, *bla*_VEB_, as well as the most commonly reported clinical subtypes of *bla*_KPC_, and *bla*_CTX-M_. Additionally all isolates were negative for all of the Group B metallo-β-lactamases (MBLs) genes tested which included, *bla*_Imp_, *bla*_VIM_, *bla*_GIM_, *bla*_SPM_, *bla*_SIM_, *bla*_DIM_, and *bla*_NDM-1_. All of the CRAB isolates tested contained the Group C cephalosporinase *bla*_ampC_ gene, while 20 (76%) also possessed the cephalosporinase *bla*_ADC-7_ gene.

### Mobile genes and insertion sequences

As described by Koeleman et al., the presence of mobile genetic elements is of particular interest as they provide strong evidence for the horizontal dissemination of antibiotic resistance genes [35]. The two mobile gene elements explored in this study were the class 1 integron cassette (ICA1) and three integrase coding genes (*int*123) as described in Hujer et al. [36]. A total of 11 (42%) of the CRAB isolates contained ICA1, and 21 (80%) of the isolates had *int*123. Only two isolates #58 (*bla*_OXA-40-like_ positive) and #64 (*bla*_OXA-23-like_ positive) from the 2010–2011 isolates were negative for both mobile gene elements, while all 10 (100%) of the 2015 CRAB isolates were positive for one or both of the mobile gene elements (Table 3). These data suggest that the majority of the CRAB isolates in present study may have ability to transfer genetic elements through integrons, and that these mobile elements may contain antibiotic resistance genes.

In addition, we detected IS elements in the promoter regions of several antibiotic resistance genes. A total of 25 (96%) of the CRAB isolates examined in this study were found to have the IS*Aba1* element upstream of OXA-51 (Additional file 5: Figure S2; Table 3), and one negative isolate #58 also carried IS*Aba1* in the non-promoter region of OXA-51 (this isolate also processes the *bla*_OXA-40-like_ gene). In addition, IS*Aba1* was found upstream of the ampC cephalosporinases in 92% of the isolates, and upstream of the ADC-7 gene in 76% of the isolates.

### Biofilm production

In vitro biofilm production assays were performed on all CRAB isolates (Fig. 1) with 9 of 26 (34%) showing varying degrees of biofilm production. Figure 1a shows the 2015 isolates includes more biofilm producers than the 2010–2011 isolates (p = 0.03). There was no significant association found between biofilm production and the source of the isolate (Fig. 1b). Evaluation of biofilm production between the two MLST strain types demonstrated that the ST-2 strains were significantly better biofilm producers than the ST-229 strains (p = 0.04) (Fig. 1c).

## Discussion

The present study was undertaken to determine whether any patterns of resistance and fitness within our clinical CRAB isolate groups evolved or expanded over time. The unique aspect to this study is the two isolate groups we used were collected 4–5 years apart. This fact allowed us to have a look longitudinally at the genotypes and phenotypes of antibiotic-resistant *A. baumannii* isolates, and to potentially identify traits that make certain isolates successful pathogens under the selective pressures of a hospital setting.

From a clinical perspective, all the isolates examined in this study displayed the MDR phenotype [34]. We aimed to find the genetic mechanisms conferring these resistances in our isolates by exploring the presence of a large number of β-lactamase encoding genes. Metallo-β-lactamase positive *A. baumannii* isolates are rare in the United States, and we did not find isolate carrying MBL gene; however globally, reports detecting their appearance are increasing in frequency [37–39]. The Group A β-lactamases encoded by *bla*_TEM_ and *bla*_SHV_ genes has been shown to hydrolyze both penicillins, as well as first- and second- generation cephalosporins [40]. Interestingly there seems to be a distinction with regard to ST type with regard to possession of the *bla*_SHV_ gene wherein the *bla*_SHV_ gene was found in 10 (83%) of ST-229 isolates, and 1 (7%) of the ST-2 isolates. Although *bla*_ampC_ and *bla*_ADC-7_ genes encode cephalosporinases were present, the later (*bla*_ADC-7_ was predominant (in 76% of isolates), which have been shown to be inducible [41, 42].

The *bla*_OXA-51-like_ allele is chromosomal and has been reported as an intrinsic gene of the *A. baumannii* species [43]. Both the *bla*_OXA-23-like_ and *bla*_OXA-40-like_ genes can be either plasmid or chromosome borne, and have an increased risk of horizontal dissemination, resulting in increased rates of resistance in healthcare settings [44, 45]. In our isolate groups, only 2 (7%) isolates contained the *bla*_OXA-40-like_ gene; interestingly these two isolates display the greatest phenotypic resistance to meropenem (≥ 256 μg/mL), while 9 (34%) of our CRAB isolates possess the *bla*_OXA-23-like_ gene, and displayed MICs between 32–64 μg/mL. These two oxacillinase genes were only found in ST-2 isolates in among the 2010–2011 isolate group; but seem to have been successfully disseminated into the hospital setting overtime as they were found in 9 (90%) of the 2015 isolate group.

A major concern in the healthcare community is the acquisition of genes such OXA-23-like and OXA-40-like through mobile genetic element transfers [46]. We did not determine whether these mobile elements such as integron-encoded integrase genes flank resistance genes, but their presence in majority (80%) of proposed CRAB suggests that those isolates are capable of acquiring and donating virulence genes by recombination.

An interesting finding from this study was the presence of IS elements upstream of several β-lactamase genes. It has been previously shown that the insertion of the IS element IS*Aba1* upstream of the OXA-51 gene changes the expression level leading to increased antimicrobial resistance [43]. Except isolate #58, which was co-carrying blaOXA-40 like gene), every isolate examined in this study was found to have IS*Aba*1 upstream of the *bla*_OXA-51-like_ gene (Additional file 5: Figure S2), and suggesting that the isolates may have additional mechanisms resistance against carbopenem. The IS*Aba1* was also present in promoter region of *bla*_ampC_ gene (92%) and *bla*_ADC-7_ gene (76%) of the isolates, and suggest the overexpression of the downstream cephalosporinase genes resulting in an enhanced resistance phenotype.

MLST typing showed differing prevalence of the two STs of resistant strains of *A. baumannii* throughout the hospital over time. A comparison suggests that the majority of the isolates of 2010–2011 group were the members of ST-229, and only 5 (32%) isolates were ST-2, whereas interestingly, 90% of the isolates of 2015 group were ST-2, and only (10%) determined as ST-229. These data indicate over the course of 4–5 years that the ST-2 has successfully replaced most of the ST-229 strains to become the predominant resistant type within our hospital.

Why is one strain type more successful than the other when it appears the two strain types share similar genetic resistance mechanisms? It has been previously reported that biofilm production is an important virulence factor for *A. baumannii* as it may aid colonization and survival on abiotic hospital environments [47]. Thus, we explored the ability of our CRAB isolates to produce biofilm on an abiotic (polystyrene) surface, to give an indication of how well these strains might colonize both medical equipment and objects in the clinical environment. We found that there were no significant differences between the clinical source of the isolates or their ability to produce biofilms (Fig. 1b). Other studies using more clonally diverse strains have shown enhanced biofilm producing isolates derived from urine and sputum cultures [48, 49]. Therefore, the non-significant result in our isolate group may be due to a lack of clonal heterogeneity. When we grouped our isolates by isolate groups (2010–2011 versus 2015) we found the 2015 isolate group had an enhanced ability to produce biofilm over the old isolates (Fig. 1a). Due to the predominance of ST-2 in the 2015 isolate group we explored biofilm production by MLST strain type, wherein the ST-2 showed an increased biofilm production compared to ST-229 (Fig. 1c). Therefore, a possible explanation for the success of ST-2 over ST-229 may be its enhanced ability to produce biofilms resulting in a more effective colonization within the hospital, even when the two strains exhibit similar antibiotic resistance phenotypes.

## Conclusion

The temporal nature of the isolates used in this study provided a unique opportunity to epidemiologically monitor multi-drug-resistant *A. baumannii* isolates from the same hospital over time. We have shown that all of the CRAB isolates in our hospital belong to either MLST strain type ST-2 or ST-229 and that these two strain types most likely share similar mechanisms of resistance with possibly IS*Aba*1-dependent overexpression of β-lactamase genes, resulting in an MDR phenotype. Interestingly over a 4–5 years’ timeframe the ST-2 type replaced the ST229 type to become the predominate CRAB strain within the hospital which we believe is due to its enhanced ability to produce biofilms leading to improved colonization and survival on abiotic surfaces. To our knowledge, this is the first study to comparatively characterize the genetic and phenotypic properties of clinical MDR *A. baumannii* isolates from a US hospital over an extended time frame.

## Additional files

**Additional file 1: Table S1.** Quality control (QC) organisms used in this study.

**Additional file 2: Table S2.** Primer sequences used for *A. baumannii* in present study.

**Additional file 3: Table S3.** Antimicrobial typing of *A. baumannii* study isolates, showing individual isolates against commonly used drugs.

**Additional file 4: Figure S1.** Multiplex PCR of CRAB isolates looking at Group D β-lactamases.

**Additional file 5: Figure S2.** PCR screening of IS*Aba*1 upstream of *ble*_OXA-51-like_ in three representative isolates.
