# Supplementary material for: Molecular characterization of β-lactamase genes in clinical isolates of carbapenem-resistant Acinetobacter baumannii
Source: Ann Clin Microbiol Antimicrob. 2017 Nov 16;16:75. doi: 10.1186/s12941-017-0248-3 (PMC5691885; doi:10.1186/s12941-017-0248-3)
Supplement: Supplementary file 1 — Additional file 1: Table S1. Quality control (QC) organisms used in this study. [file 12941_2017_248_MOESM1_ESM.pdf]

S1 Table. Quality control (QC) organisms used in this study.

**Organism (Strain ID)**	**Used for Resistance Gene (in this study)**
Acinetobacter baumannii ATCC-19606	OXA-51
Acinetobacter baumannii ATCC-17978	OXA-51
Escherichia coli ATCC-BAA-2452	NDM-1
Escherichia coli NCTC 13476	IMP
Klebsiella pneumoniae ATCC-BAA-2146	NDM-1
Klebsiella pneumoniae ATCC-BAA-1705	KPC
Klebsiella pneumoniae NCTC	VIM
Enterobacter cloacae NCTC 13464	CTX-M
