# Supplementary material for: Molecular characterization of β-lactamase genes in clinical isolates of carbapenem-resistant Acinetobacter baumannii
Source: Ann Clin Microbiol Antimicrob. 2017 Nov 16;16:75. doi: 10.1186/s12941-017-0248-3 (PMC5691885; doi:10.1186/s12941-017-0248-3)
Supplement: Supplementary file 2 — Additional file 2: Table S2. Primer sequences used for A. baumannii in present study. [file 12941_2017_248_MOESM2_ESM.pdf]

S2 Table. Primer sequences used for *A. baumannii* in present study

Primer Name	Sequence	Annealing Temp (°C)	Target Gene(s)	Reference
OXA-51-like	TAATGCTTTGATCGGCCTTG<br>TGGATTGCACTTCATCTTGC	52	$\textit{bla}_{\text{OXA-51-like}}$	1
OXA-23-like	GATCGGATTGGAGAACCAGA<br>ATTTCTGACCGCATTTCCAT	52	$\textit{bla}_{\text{OXA-23-like}}$	1
OXA-24-like	GTACTAATCAAAGTTGTGAA<br>TTCCCCTAACATGAATTTGT	50	$\textit{bla}_{\text{OXA-24-like}}$	2
OXA-40-like	GGTTAGTTGGCCCCCTTAAA<br>AGTTGAGCGAAAAGGGGATT	52	$\textit{bla}_{\text{OXA-40-like}}$	1
OXA-58-like	AAGTATTGGGGCTTGTGCTG<br>CCCCTCTGCGCTCTACATAC	52	$\textit{bla}_{\text{OXA-58-like}}$	1
IMP	GGAATAGAGTGGCTTAATTCTC<br>CCAAACCACTACGTTATCT	52	$\textit{bla}_{\text{IMP}}$	1
VIM	GATGGTGTTTGGTCGCATA<br>CGAATGCGCAGCACCAG	52	$\textit{bla}_{\text{VIM}}$	1
GIM	TCGACACACCTTGGTCTGAA<br>AACTTCCAACTTTGCCATGC	52	$\textit{bla}_{\text{GIM}}$	1
SPM	AAAATCTGGGTACGCAAACG<br>ACATTATCCGCTGGAACAGG	52	$\textit{bla}_{\text{SPM}}$	1
SIM	TACAAGGGATTCGGCATCG<br>TAATGGCCCGTTCCCATGTG	52	$\textit{bla}_{\text{SIM}}$	1
DIM	TCTATTCAGCTTGTCTTCGC<br>TGTTAGAGGCTGTCTCAGCC	52	$\textit{bla}_{\text{DIM}}$	3
NDM-1	GGTTTGGCGATCTGGTTTTC<br>CGGAATGGCTCATCACGATC	52	$\textit{bla}_{\text{NDM-1}}$	3
TEM	ATCAGCAATAAA CCAGC<br>CCCCGAAGAACGTTTTC	52	$\textit{bla}_{\text{TEM}}$	4
PER	ATGAATGTCATTATAAAAGC	50	$\textit{bla}_{\text{PER}}$	5
	AATTTGGGCTTAGGGCAGAA			
SHV	AGGATTGACTGCCTTTTTG	52	$\textit{bla}_{\text{SHV}}$	4
	ATTTGCTGATTTCGCTCG			
VEB	CAGTTTGAGCATTTGAATACAC<br>AGCGTATTTGTTGCAGAGTCC	55	$\textit{bla}_{\text{VEB}}$	5
KPC-1	GCTACACCTAGCTCCACCTTC<br>GACAGTGGTGGTAATCCATGC	64	$\textit{bla}_{\text{KPC-1}}$	6
KPC-2	GGTGATTCAGGGGTAAAGTGG<br>GACAGTGGTGGTAATCCATGC	64	$\textit{bla}_{\text{KPC-2}}$	6
KPC-5	TGCGCAACTGTTTTGTCGAG<br>CGGCATAGTCATTTGCCGTG	64	$\textit{bla}_{\text{KPC-5}}$	This study
KPC-10	TGTGTACGCGATGGATACCG<br>TCCGGTTTTGTCTCCGACTG	64	$\textit{bla}_{\text{KPC-10}}$	This study
CTX-M-1	AAAAATCACTGCGCCAGTTC<br>AGCTTATTCATCGCCACGTT	52	$\textit{bla}_{\text{CTX-M-1}}$	1
CTX-M-2	CGACGCTACCCCTGCTTT<br>CCAGCGTC GATTTTTCAGG	52	$\textit{bla}_{\text{CTX-M-2}}$	1
CTX-M-8	TCGCGTTAAGCGGATGATGC<br>AACCCACGATGTGGGTAGC	52	$\textit{bla}_{\text{CTX-M-8}}$	1

CTX-M-9	CAAAGAGAGTGCAACGGATG<br>ATTGGAAAGCGTTCATCACC	52	*bla*$_{\text{CTX-M-9}}$	1
CTX-M-25	GCACGATGACATTCGGG<br>AACCCACGATGTGGGTAGC	52	*bla*$_{\text{CTX-M-25}}$	1
ACD-7	ATGCGATTTAAAAAAATTTCTTGT<br>TTATTTCTTTATTGCATTCAG	52	*bla*$_{\text{ADC-1-7}}$	7
ampC	AGTACCTCAATTTATGCGGGCA<br>TGCATTCAGCACAGCATAAGC	52	*bla*$_{\text{ampC}}$	8
IS*Aba1*	CACGAATGCAGAAGTTG<br>CGACGAATACTATGACAC	52	IS$_{Aba1}$	9
*int*123	TGCGGGTYAARGATBTKGATTT<br>CARCACATGCGTRTARAT	64	Integrase gene (*int*$_{1\text{-}3}$)	10
ICA-1	TCATGGCTTGTTATGACTGT<br>GTAGGGCTTATTATGCACGC	55	Integron Cassette Array-1	10

References

1) 1) Woodford N. *Rapid characterization of beta-lactamases by multiplex PCR*. Methods Mol Biol 2010; 642: 181-92.

2) Bou, G., A. Oliver, and J. Martinez-Beltran. *OXA-24, a novel class D beta-lactamase with carbapenemase activity in an Acinetobacter baumannii clinical strain*. Antimicrob Agents Chemother, 2000. 44(6): p. 1556-61.

3) Poirel L, Walsh TR, Cuvillier V, Nordmann P. *Multiplex PCR for detection of acquired carbapenemase genes*. Diagn Microbiol Infect Dis 2011; 70: 119-23.

4) Colom, K., et al., *Simple and reliable multiplex PCR assay for detection of blaTEM, bla(SHV) and blaOXA-1 genes in Enterobacteriaceae.* FEMS Microbiol Lett, 2003. **223**(2): p. 147-51.

5) Naas, T., et al., *Emergence of PER and VEB extended-spectrum beta-lactamases in Acinetobacter baumannii in Belgium.* J Antimicrob Chemother, 2006. **58**(1): p. 178-82.

6) Wolter, D.J., et al., *Phenotypic and enzymatic comparative analysis of the novel KPC variant KPC-5 and its evolutionary variants, KPC-2 and KPC-4.* Antimicrob Agents Chemother, 2009. **53**(2): p. 557-62.

7) Hujer, K.M., et al., *Identification of a new allelic variant of the Acinetobacter baumannii cephalosporinase, ADC-7 beta-lactamase: defining a unique family of class C enzymes.* Antimicrob Agents Chemother, 2005. **49**(7): p. 2941-8.

8) Ben RJ, Yang MC, Hsueh JC, Shiang JC, Chien ST. *Molecular characterization of multiple drug-resistant Acinetobacter baumannii isolates in southern Taiwan*. Int J Antimicrob Agents. 2011 Nov;38(5):403-08.

9) Segal H, Garny S, Elisha BG. 2005. *Is IS(ABA-1) customized for Acinetobacter?* FEMS microbiology letters 243:425-429.

10) White, P.A., et al., *Characterisation of two new gene cassettes, aadA5 and dfrA17.* FEMS Microbiol Lett, 2000. **182**(2): p. 265-9.
