# Supplementary material for: Molecular characterization of β-lactamase genes in clinical isolates of carbapenem-resistant Acinetobacter baumannii
Source: Ann Clin Microbiol Antimicrob. 2017 Nov 16;16:75. doi: 10.1186/s12941-017-0248-3 (PMC5691885; doi:10.1186/s12941-017-0248-3)
Supplement: Supplementary file 3 — Additional file 3: Table S3. Antimicrobial typing of A. baumannii study isolates, showing individual isolates against commonly used drugs. [file 12941_2017_248_MOESM3_ESM.pdf]

S3 Table. Antimicrobial typing of *A. baumannii* study isolates, showing individual isolates against commonly used drugs.

Isolate No.	Ampicillin/Sulbact	Imipinem	Meropenem	Amikacin	Levofloxacin	Gentamicin	Cefepime	Tobramicin	Trimeth-Sulfametho	Piperacilin
22	R	R	R	SS	R	R	R	R	R	R
25	I	R	R	SS	R	R	R	R	R	R
28	SS	I	I	SS	R	SS	SS	SS	R	R
32	R	R	R	SS	R	R	R	R	R	R
39	R	R	R	SS	R	R	R	R	R	R
42	R	R	R	R	R	R	R	R	R	R
51	SS	R	R	SS	R	R	R	R	R	SS
52	SS	R	R	SS	R	R	R	R	R	I
56	R	R	R	R	R	R	R	R	R	R
57	I	I	R	SS	R	R	R	SS	R	R
58	R	R	R	R	I	R	R	R	R	R
61	R	R	R	R	R	R	R	R	SS	
62	R	R	R	R	R	R	R	R	R	R
64	SS	R	R	R	R	R	R	R	R	R
65	SS	R	R	R	R	R	R	R	R	R
68	I	R	R	R	R	R	R	SS	R	R
220	SS	R	R	R	R	SS	I	SS	R	R
222	I	R	R	R	R	R	R	SS	R	R
223	I	R	R	R	R	R	R	SS	R	R
224	SS	R	R	R	R	R	R	I	R	R
225	I	R	R	R	R	SS	R	SS	SS	R
226	SS	R	R	I	R	SS	I	SS	R	R
229	SS	R	R	R	R	R	R	SS	R	R
231	I	R	R	R	R	R	R	SS	R	R
232	I	R	R	R	R	R	R	SS	R	R
235	I	R	R	I	R	SS	R	SS	R	R
