# Supplementary figures and images for: Molecular characterization of β-lactamase genes in clinical isolates of carbapenem-resistant Acinetobacter baumannii
Source: Ann Clin Microbiol Antimicrob. 2017 Nov 16;16:75. doi: 10.1186/s12941-017-0248-3 (PMC5691885; doi:10.1186/s12941-017-0248-3)

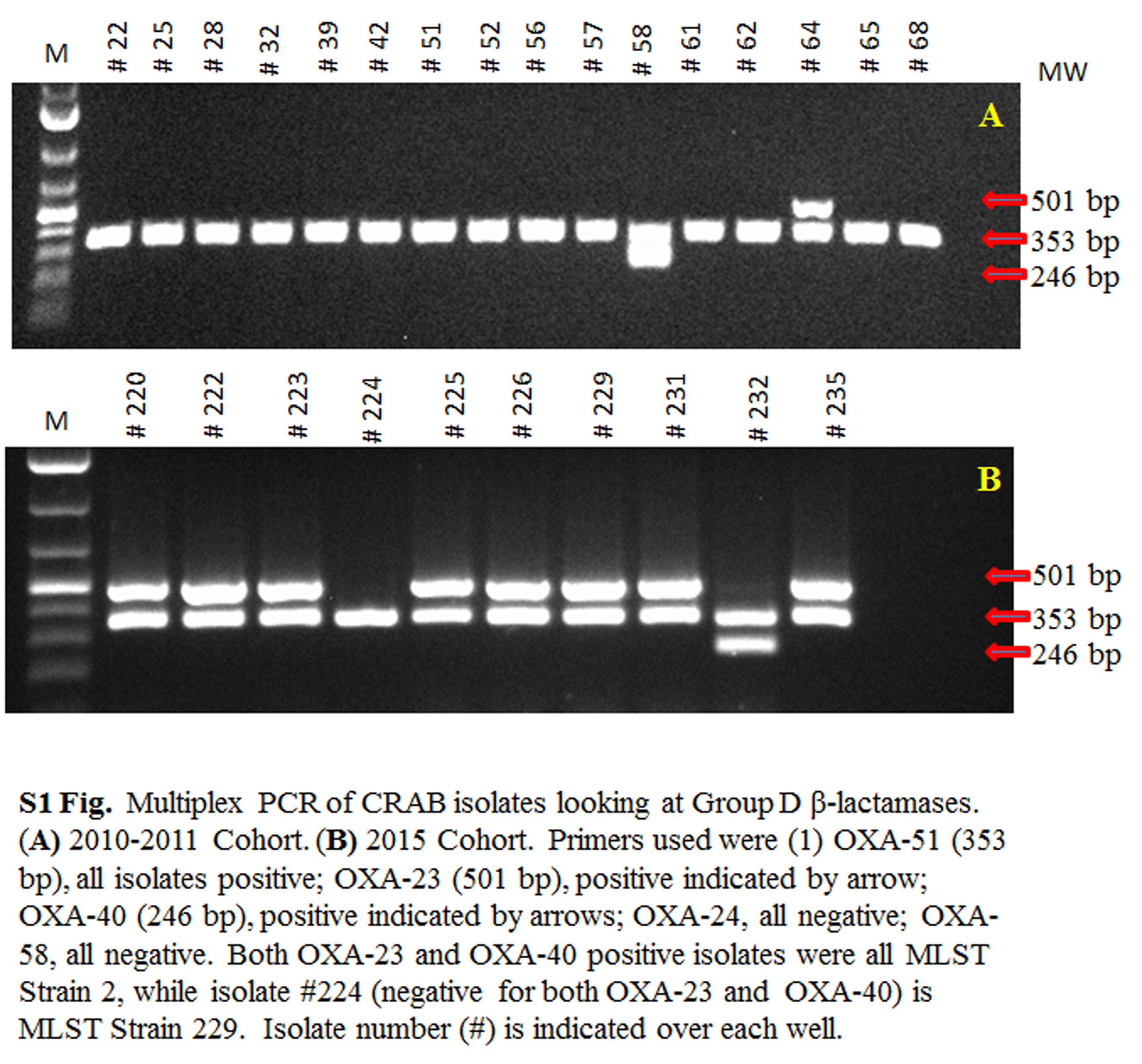

Supplement: Supplementary file 4 — Additional file 4: Figure S1. Multiplex PCR of CRAB isolates looking at Group D β-lactamases. [file 12941_2017_248_MOESM4_ESM.tif]

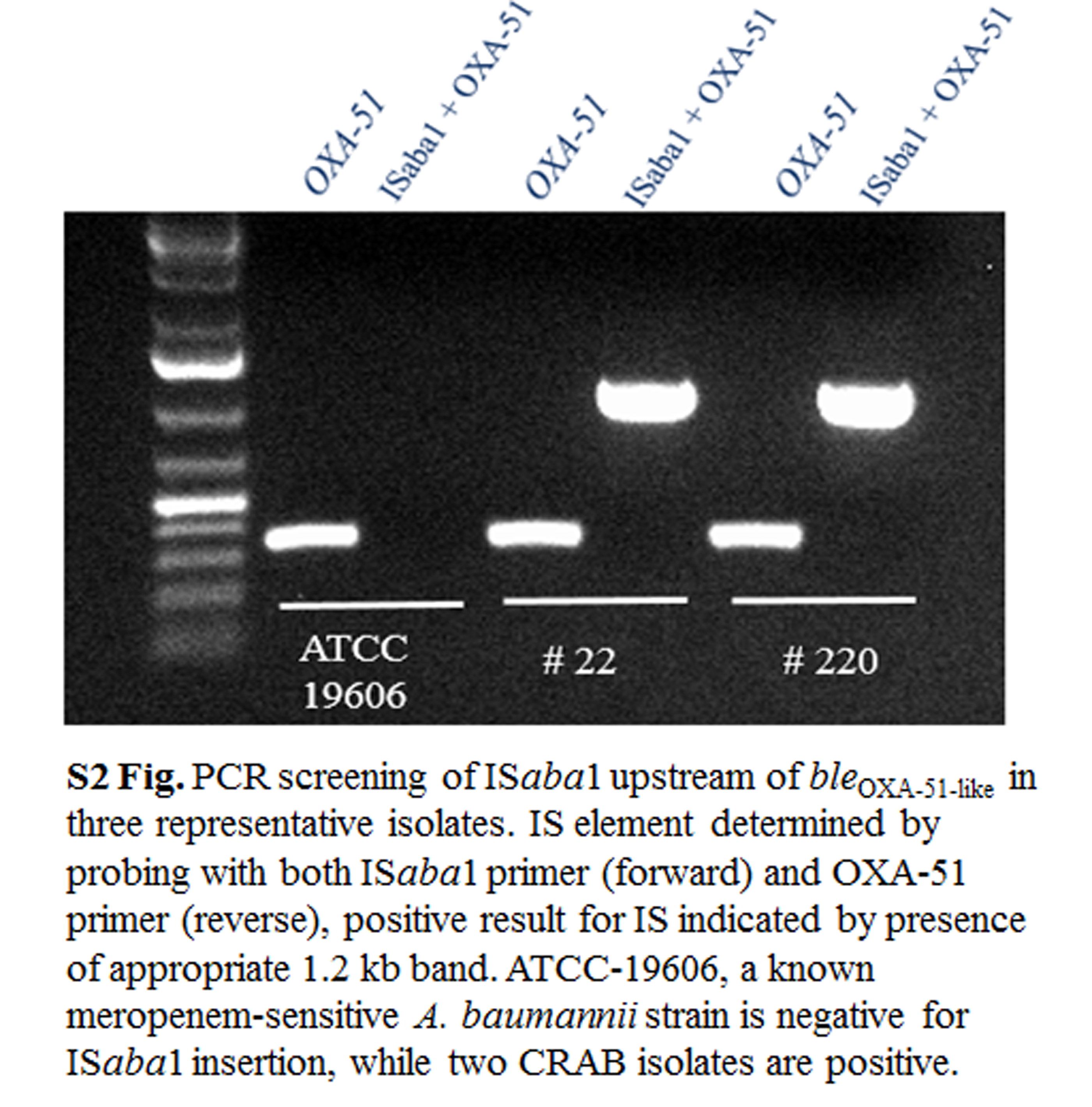

Supplement: Supplementary file 5 — Additional file 5: Figure S2. PCR screening of ISAba1 upstream of ble OXA-51-like in three representative isolates. [file 12941_2017_248_MOESM5_ESM.tif]
